# Multifunctional Polymeric Biodegradable and Biocompatible Coatings Based on Silver Nanoparticles: A Comparative In Vitro Study on Their Cytotoxicity towards Cancer and Normal Cell Lines of Cytostatic Drugs versus Essential-Oil-Loaded Nanoparticles and on Their Antimicrobial and Antibiofilm Activities

**DOI:** 10.3390/pharmaceutics15071882

**Published:** 2023-07-04

**Authors:** Rebecca Alexandra Puiu, Alexandra Cătălina Bîrcă, Valentina Grumezescu, Liviu Duta, Ovidiu Cristian Oprea, Alina Maria Holban, Ariana Hudiță, Bianca Gălățeanu, Paul Cătălin Balaure, Alexandru Mihai Grumezescu, Ecaterina Andronescu

**Affiliations:** 1 Department of Science and Engineering of Oxide Materials and Nanomaterials, Politehnica University of Bucharest, 011061 Bucharest, Romania; rebecca_alexandra92@yahoo.com (R.A.P.); ada_birca@yahoo.com (A.C.B.); grumezescu@yahoo.com (A.M.G.); ecaterina.andronescu@upb.ro (E.A.); 2 Lasers Department, National Institute for Lasers, Plasma and Radiation Physics, 409 Atomistilor Street, 077125 Magurele, Romania; valentina_grumezescu@yahoo.com (V.G.); liviu.duta@inflpr.ro (L.D.); 3 Department of Inorganic Chemistry, Physical Chemistry and Electrochemistry, Politehnica University of Bucharest, 011061 Bucharest, Romania; ovidiu73@yahoo.com; 4 Academy of Romanian Scientists, Ilfov No. 3, 050044 Bucharest, Romania; 5 Department of Microbiology and Immunology, Faculty of Biology, University of Bucharest, 91-95 Splaiul Independentei Street, 077206 Bucharest, Romania; alina_m_h@yahoo.com; 6 Department of Biochemistry and Molecular Biology, University of Bucharest, 050095 Bucharest, Romania; arianahudita@yahoo.com (A.H.); bianca.galateanu@bio.unibuc.ro (B.G.); 7 Department of Organic Chemistry, Politehnica University of Bucharest, 011061 Bucharest, Romania; 8 Research Institute of the University of Bucharest—ICUB, University of Bucharest, 050657 Bucharest, Romania

**Keywords:** cancer therapy, silver nanoparticles, MAPLE, essential oil, nanocoatings, cytostatic drugs

## Abstract

We report on a comparative in vitro study of selective cytotoxicity against MCF7 tumor cells and normal VERO cells tested on silver-based nanocoatings synthesized by the matrix-assisted pulsed laser evaporation (MAPLE) technique. Silver nanoparticles (AgNPs) were loaded with five representative cytostatic drugs (i.e., doxorubicin, fludarabine, paclitaxel, gemcitabine, and carboplatin) and with five essential oils (EOs) (i.e., oregano, rosemary, ginger, basil, and thyme). The as-obtained coatings were characterized by X-ray diffraction, thermogravimetry coupled with differential scanning calorimetry, Fourier-transform IR spectroscopy, IR mapping, and scanning electron microscopy. A screening of the impact of the prepared nanocoatings on the MCF7 tumor and normal VERO cell lines was achieved by means of cell viability MTT and cytotoxicity LDH assays. While all nanocoatings loaded with antitumor drugs exhibited powerful cytotoxic activity against both the tumor and the normal cells, those embedded with AgNPs loaded with rosemary and thyme EOs showed remarkable and statistically significant selective cytotoxicity against the tested cancercells. The EO-loaded nanocoatings were tested for antimicrobial and antibiofilm activity against *Staphylococcus aureus*, *Escherichia coli*, and *Candida albicans*. For all studied pathogens, the cell viability, assessed by counting the colony-forming units after 2 and 24 h, was significantly decreased by all EO-based nanocoatings, while the best antibiofilm activity was evidenced by the nanocoatings containing ginger and thyme EOs.

## 1. Introduction

Behind cardiovascular diseases (CVDs), cancer is the second leading cause of death, accounting for nearly 10 million deaths worldwide in 2020 [1]. Moreover, an epidemiological study published in *The Lancet* claimed that despite CVDs remaining the main cause of death globally, in some high-income countries (HICs), it has been overtaken by cancer. More specifically, the ratio of cardiovascular to cancer deaths was 0.4 in HICs, 1.3 in middle-income countries (MICs), and 3.0 in low-income countries (LICs) [2]. These results mark an epidemiological transition that can be partly explained by the successful implementation of specific long-term programs and strategies aimed at preventing and treating CVDs in HICs. It should be mentioned that achieving an effective therapy of the various types of cancer is severely hampered by a series of major drawbacks of the conventional chemotherapeutic agents, such as reduced cell membrane permeability, low accumulation into the tumor tissues, lack of targeted delivery, reduced solubility, and dose-dependent toxicity. All these could generate serious harmful side effects [3].

All the above drawbacks could be mitigated or avoided with the aid of nanotechnology. Due to their dimensions in the nano range, nanoparticles used as drug carriers can penetrate biological barriers and interact with their biological targets much more effectively than bulk materials. Proper surface functionalization can improve both the solubility and stability of the nanocarrier in biological fluids, while its conjugation with active targeting ligands guides the nanovehicle to the diseased tissue through specific cell receptors—ligand supramolecular interactions [4,5,6,7,8,9]. The antitumor chemotherapeutic agent can be either embedded into the nanoparticle or attached to the surface through physical interactions or covalent conjugation [10,11]. The nanocarrier structure can be designed to release its therapeutic payload in a sharp response to either internal (pH, temperature, redox potential, or enzyme activity) or external (light of specific wavelength, ultrasounds, or magnetic or electric field) stimuli [12,13,14,15,16,17,18,19]. The controlled release process can be triggered either by altering the nanocarrier structural integrity or by the cleavage of the covalent bond. Several therapeutic strategies, such as photodynamic therapy (PDT) [20], sonodynamic therapy (SDT) [21], magnetic hyperthermia [22,23], and chemodynamic therapy (CDT) [24,25], have been developed to reduce significantly the severe side effects associated with the conventional systemic delivery of chemotherapeutics.

Silver nanoparticles (AgNPs) exhibit intrinsic antimicrobial and anti-tumoral activity due to an enhanced generation of reactive oxygen species (ROS) as a consequence of their increased chemical reactivity. Enhanced ROS production induces DNA damage, apoptosis, and cell cycle arrest and inhibits cell proliferation [26,27,28]. Remarkable effects of AgNPs against different cancer cell lines, such as cervical adenocarcinoma cell line (HeLa), colorectal cancer (HCT-116), breast cancer (MCF7), and liver cancer (HepG2), were reported [29].

Studies showed successful combinations between AgNPs and chemotherapeutic drugs [30,31,32]. One example of an anti-cancer drug is paclitaxel, which has a cytotoxic effect on the osteosarcoma cell line by targeting specific cancer cells without causing cytotoxicity on the normal ones [33].

Although cytostatic drugs, such as doxorubicin, carboplatin, paclitaxel, gemcitabine, or fludarabine, show an important clinical response during treatment, current cancer therapies present a series of important disadvantages, such as cancer recurrence, drug resistance, and lack of selective cytotoxicity, which damage healthy tissues and cause numerous harmful side effects [34]. Therefore, natural substances presenting an increased biological activity can be an alternative solution. EOs found in aromatic plants present many remarkable properties—antioxidant, antimutagenic, and antiproliferative—and can be a suitable solution to conventional cancer treatment [35]. Due to the many functional groups in their chemical structure, one can assume that EOs can work as an adjuvant and increase the sensitivity of cancerous cells to chemotherapy by reducing metastasis and angiogenesis [36]. It was also shown that components of thyme and origanum EOs showed good pharmacological and anti-tumoral effects, acting as a good controller of pain for cancer patients [37,38].

The aim of the present study was to comparatively assess the cytotoxic activity against MCF7 tumor and healthy VERO cell lines of classic cytostatic/natural-EO-loaded AgNPs embedded in a biodegradable and biocompatible polymeric matrix obtained by MAPLE for anti-tumoral applications. In addition, the antimicrobial and antibiofilm activity of the prepared nanocoatings against representative model bacterial and fungal strains, namely *Staphylococcus aureus* (*S. aureus*), *Escherichia coli* (*E. coli*), and *Candida albicans* (*C. albicans*), was also tested.

## 2. Materials and Methods

### 2.1. Materials

Chemical substances used to synthesize AgNPs were purchased from Sigma-Aldrich (Merck Group, Darmstadt, Germany): Silver Nitrate (AgNO_3_), D-Glucose, Sodium Hydroxide (NaOH), Paclitaxel, Doxorubicin, Fludarabine, Gemcitabine, Carboplatin, Chloroform (CHCl_3_), and poly(3-hydroxybutyrate-co-3-hydroxyvalerate) natural origin, PHBV content 8 mol % (PHBV). EOs—oregano, rosemary, ginger, basil, and thyme—were purchased from Solaris. All reagents were of analytical purity and used without further purification.

### 2.2. Chemical Synthesis of AgNPs Loaded with Cytostatic and Essential Oils

AgNPs were prepared by the bottom-up chemical reduction method. In brief, solution 1 (Sn1) was prepared by dissolving 0.5 g AgNO_3_ in 100 mL of distilled water. Solution 2 (Sn2) consisted of 1 g D-glucose and 4 g NaOH dissolved in 400 mL distilled water at the temperature of 80 °C under magnetic stirring. In the next step, Sn1 was added dropwise using a pressure-equalizing dropping funnel into Sn2 under magnetic stirring. In the case of AgNPs functionalized with cytostatic agents, 100 mg of doxorubicin, fludarabine, carboplatin, gemcitabine, and paclitaxel were added to Sn2, and in the case of AgNPs functionalized with EOs, 200 µL of rosemary, basil, thyme, oregano, and ginger were added to Sn2. At this step, the obtained precipitate was collected, and after decantation of the liquid phase, the resulting powder was washed three times with demineralized water and dried at room temperature (RT). Nanoparticles were characterized by X-ray powder diffraction (XRD), scanning electron microscopy (SEM), thermogravimetric analysis (TGA), and Fourier-transform infrared (FTIR) spectroscopy.

Table 1 introduces the sample codes of all the materials used in this paper.

### 2.3. MAPLE Deposition of the PHBV/AgNPs Nanocoatings

For the MAPLE experiments, a KrF* excimer laser source (λ = 248 nm, τ_FWHM_ ≤ 25 ns), operated at a repetition rate of 15 Hz, was used. For the synthesis of multifunctional coatings, the composite targets were fabricated by freezing in liquid-nitrogen-blended solutions with a concentration of 2 wt.%. The organic–inorganic blends were obtained by mixing the PHBV/AgNPs pristine (used as control) and PHBV/AgNPs with five types of anti-tumoral and five types of EOs, in a 3:1 ratio, followed by their suspension in chloroform. The composite coatings were grown onto glass and 100 silicon wafers, with dimensions of 10 × 10 mm^2^.

The experiments were performed at a residual pressure of 1 Pa, at RT, and a target-to-substrate separation distance of 5 cm. For each coating, 35,000 consecutive laser pulses were applied. To identify the optimal parameters to preserve the chemical structure and to diminish the absorption of laser wavelength by the concerned materials, a fluence parametric study (i.e., 200, 300, 400, 500, and 600 mJ/cm^2^) was performed. To obtain uniform coatings and avoid drilling, both target and substrates were rotated during the deposition process. Moreover, to accurately control the energy distribution onto the target surface, a laser beam homogenizer was used. The as-obtained coatings were subjected to physical–chemical characterization and biological assays.

### 2.4. Physical-Chemical Characterization

AgNPs were investigated to determine their crystallinity by XRD analysis using a diffractometer Shimadzu XRD 6000. The patterns of X-ray diffraction were recorded using CuKα radiation (λ = 1.54056 Å at 15 mA and 30 kV) at room temperature, with a Bragg diffraction angle 2θ between a range of 10 to 80°.

The nanometric details, size, and shape of the surface of functionalized AgNPs probes were investigated by SEM analyses using a FEI electron microscope with 30 keV energy of secondary electron beams.

IR Mapping (IRM) was recorded using a Nicolet iN10 MX FT-IR Microscope with MCT liquid nitrogen cooled detector, ranging between 4000 and 1000 cm^−1^. The spectral collection was made at 4 cm^−1^ resolution in reflection mode. Using OmincPicta software (Thermo Scientific, Waltham, MA, USA), 32 scans were added and converted to absorbance. For each probe, approximately 250 spectra were analyzed.

Thermal analyses were performed using a Netzsch STA 449C F3 instrument, the temperature ranging between RT and 900 °C with a heating rate of 10 K min^−1^, under dried synthetic air with a flow of 50 mL min^−1^ (80% N_2_ and 20% O_2_).

### 2.5. Biological Evaluation

#### 2.5.1. In Vitro Cell Culture Models

To investigate the cytotoxic potential of the original PHBV/AgNPs coatings embedded with anticancer drugs or natural compounds, one normal and one tumor cell line were employed. VERO (ATCC CCL-81™) and MC7 cell lines (ATCC HTB-22™) were cultured in Eagle’s Minimum Essential Media (EMEM, Sigma Aldrich, Merck Group, Darmstadt, Germany), supplemented with 10% fetal bovine serum (FBS, Life Technologies, Foster City, CA, USA) and 1% penicillin/streptomycin mixture (10,000 units/mL penicillin and 10 mg/mL streptomycin) and maintained in standard cell culture conditions (37 °C, 5% CO_2_) throughout the experiments. Additionally, as recommended by ATCC, the culture media of MCF7 cells was enriched before use with 0.01 mg/mL human recombinant insulin.

Before cell seeding, all experimental samples were subjected to UV light sterilization before transfer to 12-well cell culture plates in aseptic conditions. Both normal and tumor cells were seeded in drops on each sample surface at an initial density of 3 × 10^4^ cells/cm^2^ and left for 2 h to adhere before immersing samples in complete culture media. All samples were maintained in a 5% CO_2_ humidified atmosphere at 37 °C for 5 days. During each day, various cell health parameters were investigated to screen the cytotoxic potential of the original PHBV/AgNPs coatings.

#### 2.5.2. In Vitro Cytotoxicity Screening

After 2 and 5 days of culture, the metabolic cell health of both normal and tumor cells was investigated using the MTT assay to highlight the impact of drug/natural-compound-loaded PHBV/AgNPs compounds on cell survival rates. Briefly, at each time point, the cell culture media was discarded, and the samples were immersed for 4 h at 37 °C in a freshly prepared solution of MTT (1 g/mL) in the dark. The resulting formazan crystals were solubilized by DMSO (Sigma Aldrich), and the optical density (OD) of the resulting solutions was determined at 550 nm with the microplate reader FlexStation III (Molecular Devices, San Jose, CA, USA). The percentage of the surviving cells was calculated with the following formula: % viability = (100 × (A_550nm_ control − A_550nm_ sample)), using the corrected absorbances obtained after subtracting the culture media background.

LDH assay was performed using the “In vitro toxicology assay kit lactate dehydrogenase based TOX—7” kit (Sigma Aldrich) that measures the amount of enzyme released in the culture media by damaged cells in response to material contact. In this view, culture media was collected from all samples and mixed with the kit’s components according to the manufacturer’s recommendations. The OD of the resulting solution was determined at 490 nm at the microplate reader FlexStation III. The percentage of cytotoxicity was calculated as the ratio (A_490nm_) of treated cells to control ones.

All the final data obtained during the cytotoxicity screening represented the mean ± standard deviation (SD) of three independent experiments. For statistical analyses, GraphPad Prism software (San Diego, CA, USA) was employed, with significant level *p* < 0.05.

#### 2.5.3. Cell Morphology Investigation

To evaluate the impact of normal and tumor cells’ contact with drug-loaded PHBV/AgNPs coatings on cell morphology, fluorescence microscopy was employed. In this view, cell culture media was discarded, and samples were immersed in a 4% paraformaldehyde solution (PFA, Sigma Aldrich) for 20 min for cell fixation. Subsequently, for permeabilization, the PFA solution was replaced with a 2% BSA solution enriched with 0.1% Triton X100. Then, samples were stained with fluorescein isothiocyanate (FITC) conjugated phalloidin for 1 h at 37 °C and 20 min with 4,6-diamidino-2-phenylindole (DAPI). After staining, samples were analyzed using the Olympus IX73 fluorescent microscope (Olympus Life Science, Waltham, MA, USA), and images were captured and processed with CellSense F software (version 1.11).

#### 2.5.4. Antimicrobial Efficiency

In order to evaluate the in vitro antimicrobial activity of the obtained coatings, we analyzed the growth and viability of planktonic cultures and biofilms. One Gram-positive (i.e., *S. aureus* ATCC 23235) model, one Gram-negative (i.e., *E. coli* ATCC 25922) model, and one yeast (i.e., *C. albicans* ATCC 10231) strain were used in this study.

##### Bacterial Viability

Overnight bacterial cultures of the microbial strains were used to prepare microbial suspensions of 0.5 McFarland (approximately 1.5 × 10^8^ CFU/mL) density in sterile phosphate-buffered saline (PBS). The prepared coatings were sterilized by UV irradiation for 20 min before analysis. Then, they were added into sterile 24 multi-well plates containing 1 mL of nutritive broth, and 10 µL of the obtained suspension was seeded in each sample. The plates were incubated for 2 h and 24 h at 37 °C in a moist atmosphere. For the viability cell assay, after the incubation time, the coating sample was resuspended in 1 mL PBS into an Eppendorf tube to recover the viable microbial cells. Tubes were vigorously vortexed for 20 s to release the microbial cells into the suspension. Then, 30 µL of the obtained suspensions were transferred to 96-well plates containing 270 µL of PBS. Serial dilutions were obtained from each sample and then cultured in triplicate on nutrient agar plates for 24 h at 37 °C. The obtained colonies were then counted, and the CFU/mL (colony-forming units/mL) was achieved [39].

##### Biofilm Development

To evaluate the formation of bacterial biofilm, the obtained coatings based on AgNPs were equally distributed on 24-well plates in 1 mL nutritive broth, each being experimentally inoculated with 10 μL of the 0.5 McFarland standard density of microbial suspension. After this, the samples were incubated for 24 h at 37 °C to allow biofilm development. After incubation, the samples were washed with PBS to remove unattached cells and transferred in 1 mL PBS in a sterile tube. Then, the tubes containing the 24 h biofilms developed on the coatings were vigorously vortexed for 20 s and subjected to ultrasound treatment to detach the biofilm cells. The suspensions containing detached biofilm cells were subjected to serial 10-fold dilutions, and each dilution was inoculated on nutritive agar in triplicate for viable count analysis, expressed as CFU/mL (colony-forming units/mL).

The statistical significance (* *p* ≤ 0.05, ** *p* < 0.001) was determined using the non-parametric two-way ANOVA algorithm and Bonferroni test.

## 3. Results

### 3.1. Physical-Chemical Characterization of AgNPs

**Figure 1:**
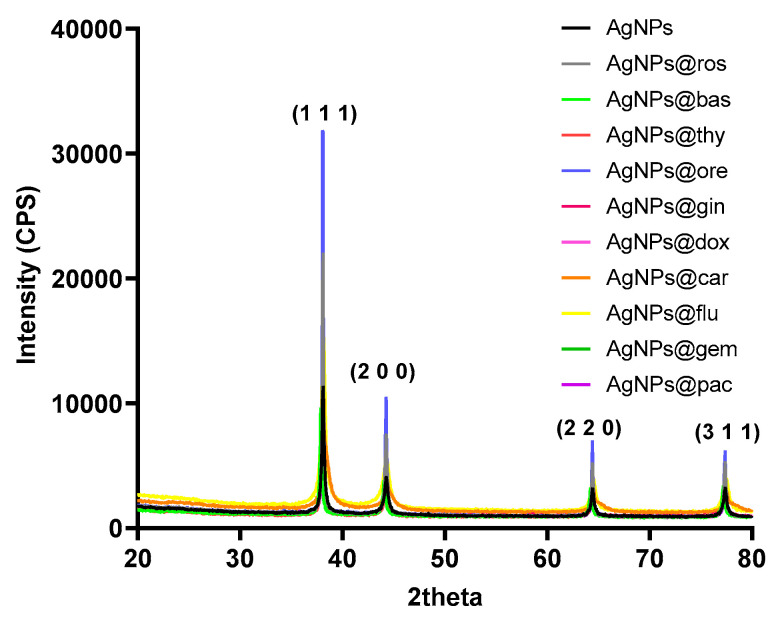
XRD patterns of AgNP powders.

The XRD patterns of the AgNPs can be observed in Figure 1. Four diffracted intensities in the range 20°–80° were measured. The XRD patterns confirm the cubic crystal structure of the AgNPs. Specific peaks were identified at 2θ values of 38.45°, 46.35°, 64.75°, and 78.05°, which correspond to the (1 1 1), (2 0 0), (2 2 0), and (3 1 1) diffraction plans of the AgNP crystals (JCPDS (00-001-1167) [40].

**Figure 2:**
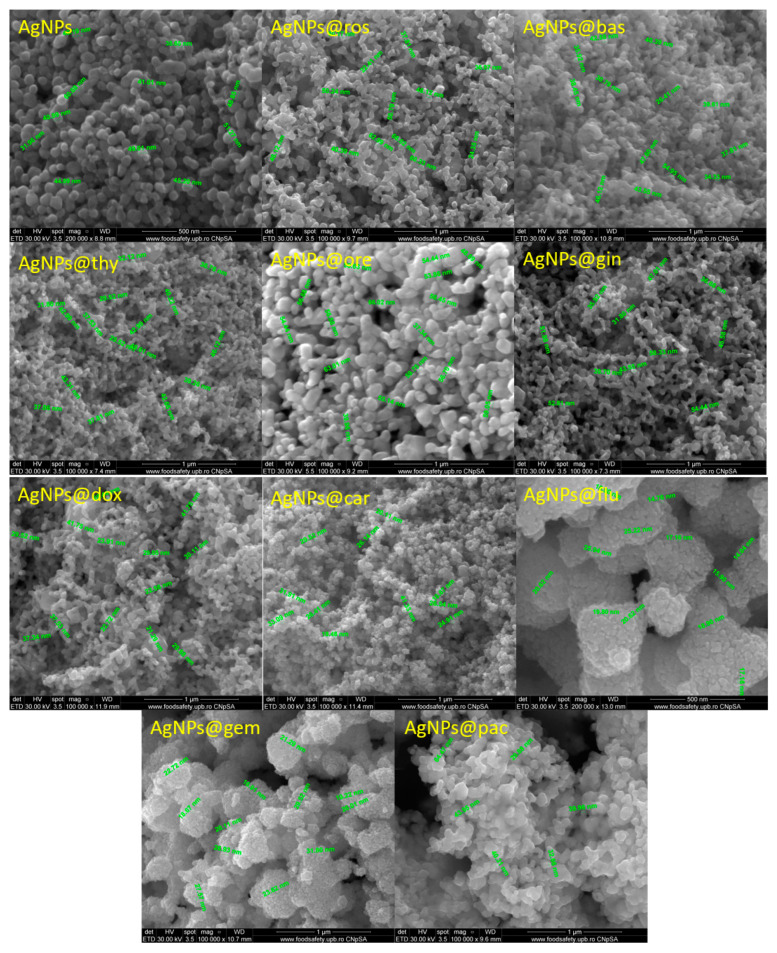
SEM images of AgNPs functionalized with different cytostatic drugs and essential oils.

The SEM micrographs (Figure 2) show the nanometric size of the AgNPs. The particles have a quasi-spherical shape, with diameters varying from 20 to 60 nm. In addition, it can be observed that the AgNPs functionalized with EOs have higher diameters than those functionalized with cytostatic drugs. Most likely, this phenomenon is due to the complexity of the EOs that contain hundreds of compounds, while cytotstatic drugs are composed of single molecules.

**Figure 3:**
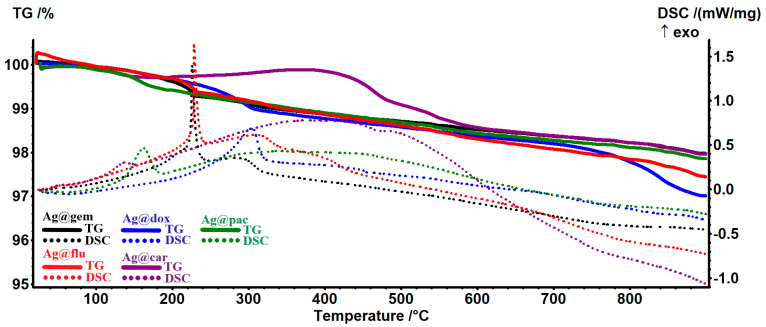
The TG-DSC curves for the samples of AgNPs loaded with cytostatic drugs.

The AgNP sample presents two small exothermic peaks—at 164 °C due to oxidation of the D-glucose and at 442 °C due to the burning of carbonaceous residual mass—the sample at 900 °C being formed only from white silver powder. The samples loaded with cytostatic drugs present a similar thermal behavior, depicted in Figure 3. The principal numeric data are presented in Table 2. The organic parts from Ag@gem and Ag@flu contain a pentose cycle and are oxidized at 226–229 °C, as indicated by the strong, sharp exothermic effect on the DSC curve. For the other three samples, the oxidation process is slower, with the exothermic peak being broader and having a much lower intensity. In the case of the Ag@car sample, a different TG curve can be observed due to the platinum presence; this will remain in the sample, unlike the purely organic drugs.

**Figure 4:**
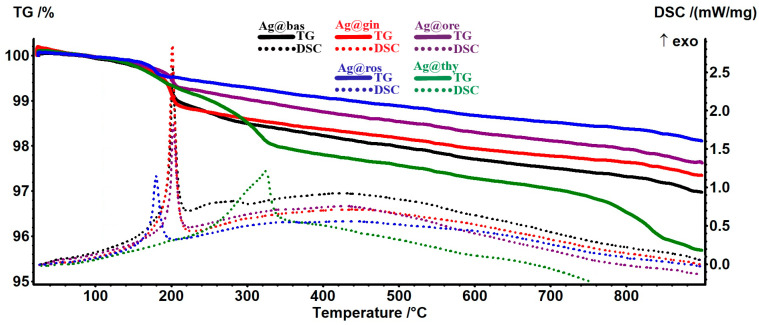
The TG-DSC curves for the samples of AgNPs loaded with EOs.

As many components are similar in the EOs, their thermal behaviors are also similar (Figure 4). The AgNPs loaded with EOs present a mass loss step at approximately 190–210 °C for Ag@bas, Ag@gin, and Ag@ore; between 170 and 210 °C for Ag@ros; and between 210 and 360 °C for Ag@thy. These mass loss steps correspond to the oxidation of the most susceptible components from the EOs. For the Ag@bas, Ag@gin, and Ag@ore samples, the exothermic effect is consistent at ~202 °C, indicating the same class of compounds that are oxidized. The burning of the residual carbonaceous mass takes place at higher temperatures. The silver-white residual mass values are presented in Table 3, together with the estimated EOs loads.

### 3.2. Physical-Chemical Characterization of Multifunctional Coatings

#### 3.2.1. Characterization of Control Sample Coatings

**Figure 5:**
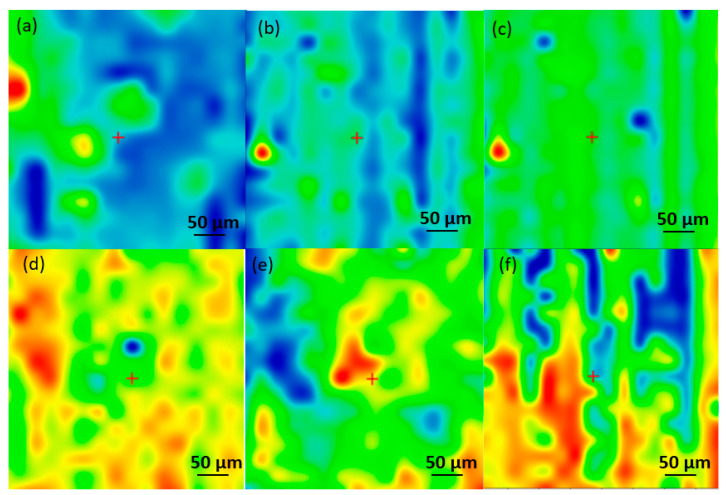
Infrared micrographs of PHBV/AgNPs obtained based on the intensity of C-H for (**a**) drop-cast and coatings at laser fluences of (**b**) 200 mJ/cm^2^; (**c**) 300 mJ/cm^2^; (**d**) 400 mJ/cm^2^; (**e**) 500 mJ/cm^2^; and (**f**) 600 mJ/cm^2^.

**Figure 6:**
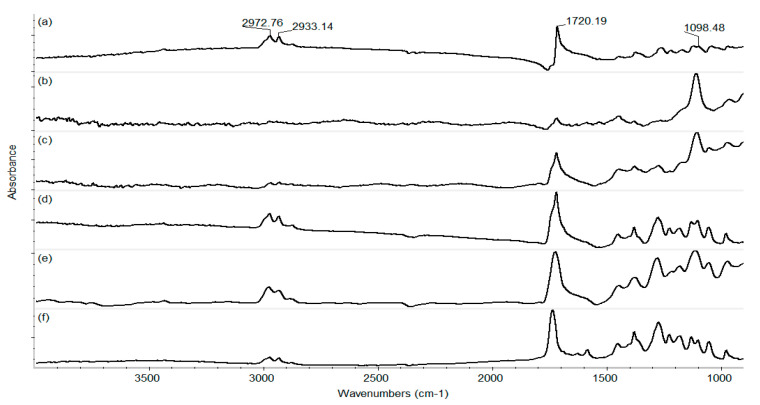
IR spectra of PHBV/AgNPs for (**a**) drop-cast and coatings at laser fluences of (**b**) 200 mJ/cm^2^; (**c**) 300 mJ/cm^2^; (**d**) 400 mJ/cm^2^; (**e**) 500 mJ/cm^2^; and (**f**) 600 mJ/cm^2^.

To investigate the compositional integrity of the control materials, comparative infrared studies (Figure 5 and Figure 6) were conducted between the drop-cast (DC) sample and the coatings obtained by MAPLE. Figure 5 presents the IR maps obtained based on the intensity absorbance of the C-H group characteristic of the PHBV. As a general remark regarding the IR microscopy analysis, the absorbance intensity of the collected infrared spectra is directly related to the color changes within the resulting IR maps, ranging from blue to red (corresponding to the lowest and highest intensities, respectively). The IR analysis (Figure 6a) shows that the DC sample with absorption bands characteristic of PHBV (C-H, C=O, and C-O) is confirmed. Compared with the IR spectra of 200 and 300 mJ/cm^2^, the C-H group has a low intensity, or it is missing, and it begins to be available at 400, 500, and 600 mJ/cm^2^. The peak intensity is similar to that of the DC sample, only at 400 mJ/cm^2^.

**Figure 7:**
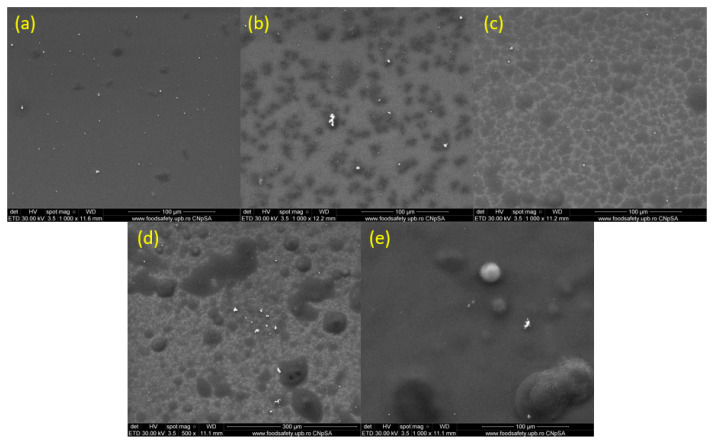
SEM images of PHVB/AgNPs coatings at 1000× (**a**–**e**), obtained at laser fluences of (**a**) 200 mJ/cm^2^; (**b**) 300 mJ/cm^2^; (**c**) 400 mJ/cm^2^; (**d**) 500 mJ/cm^2^; and (**e**) 600 mJ/cm^2^.

Figure 7 presents micrographs of surface nanocoatings fabricated at laser fluences ranging from 200 to 600 mJ/cm^2^ (Figure 7a–e). According to Figure 7, the surfaces are well-covered with PHBV/AgNPs. In all samples, the surfaces present agglomerates consisting of embedded materials well dispersed in the polymer matrix. An increased uniformity of the surface could be observed in the case of the nanocoatings obtained at a laser fluence of 400 mJ/cm^2^ (Figure 7c).

**Figure 8:**
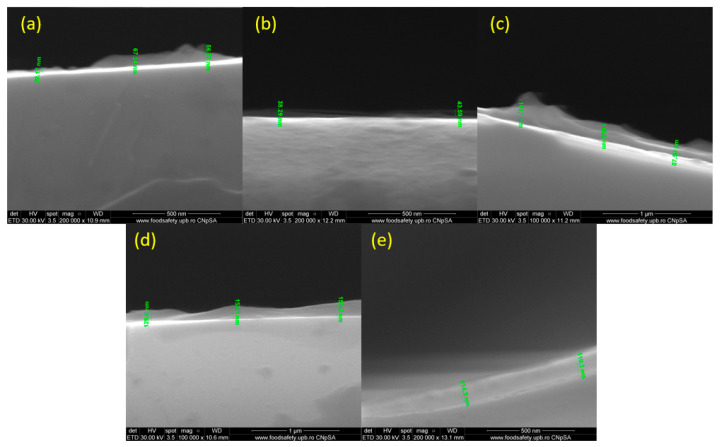
Cross-section of PHBV/AgNPs coatings at laser fluences of (**a**) 200 mJ/cm^2^; (**b**) 300 mJ/cm^2^; (**c**) 400 mJ/cm^2^; (**d**) 500 mJ/cm^2^; and (**e**) 600 mJ/cm^2^.

Figure 8 depicts the thickness of the nanostructured coatings obtained at 200, 300, 400, 500, and 600 mJ/cm^2^ laser fluences. As can be seen, the thickness varies between 30 and 150 nm, which delineates the small agglomeration tendency.

#### 3.2.2. Characterization of Optimized Composite Coatings

**Figure 9:**
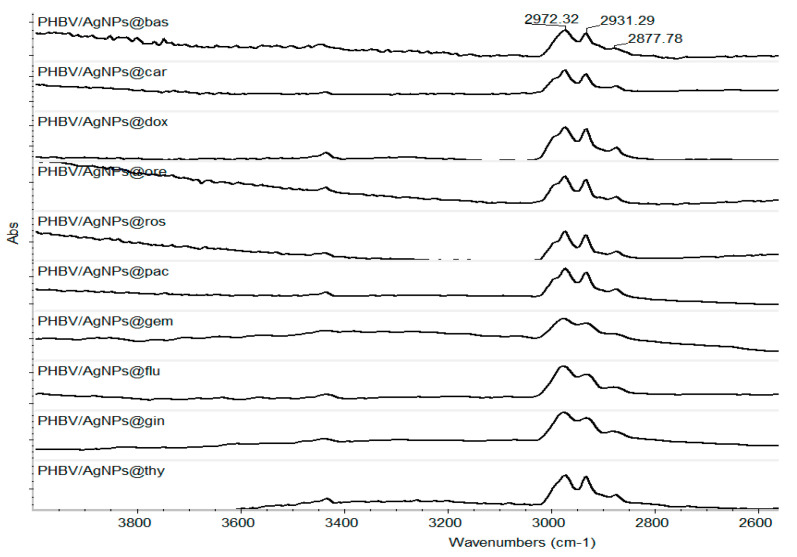
IR spectra of the coatings obtained at 400 mJ/cm^2^ laser fluence.

Taking into consideration the physical–chemical results obtained for the control coatings, the laser fluence of 400 mJ/cm^2^ was considered the optimum value to continue our investigations. Thus, Figure 9 presents the IR spectra of all composite coatings. One can observe, as already stated, that there is no degradation of the C-H group. All samples have the complete absorbance characteristics of this group.

**Figure 10:**
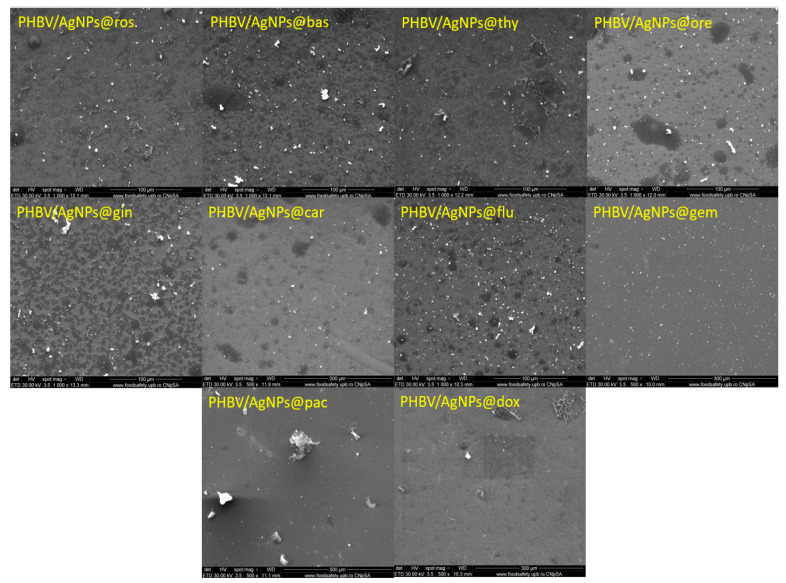
SEM images of the coatings (500–1000×) obtained at a laser fluence 400 mJ/cm^2^.

The morphological characteristics of all composite coatings can be observed in Figure 10. The coatings had a uniform deposition, and some agglomerates were present on the surface.

### 3.3. Biological Evaluation of Multifunctional Bio-Coatings Based on Silver Nanoparticles

#### 3.3.1. In Vitro Cytotoxicity Screening of PHBV/AgNPs Coatings Embedded with Anticancer Drugs or Natural Compounds

**Figure 11:**
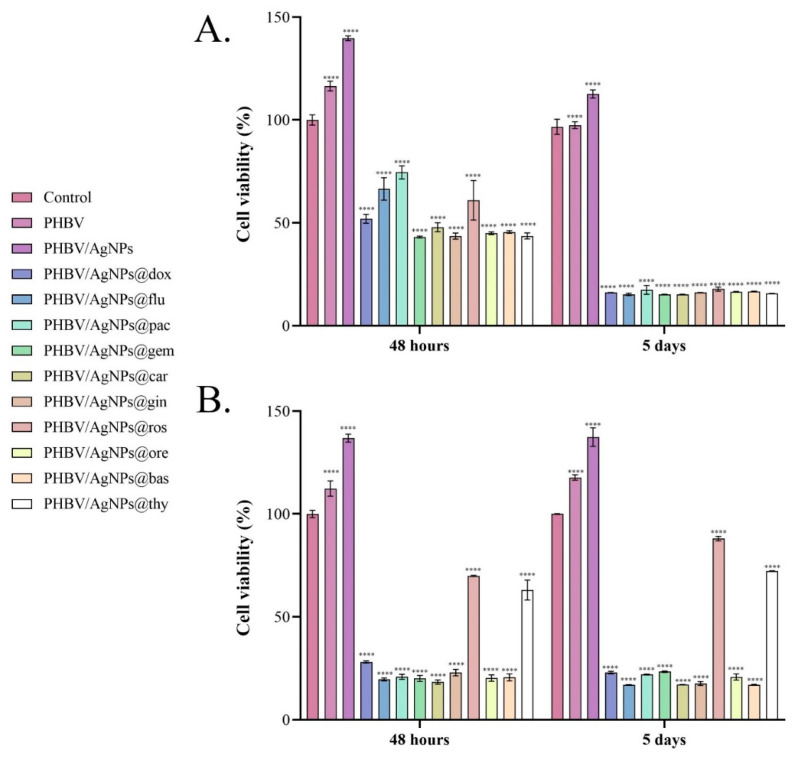
Graphical representation of cell viability of (**A**) MCF7 tumor cells and (**B**) VERO cells after 48 h and 5 days of contact with uncoated samples (control) and PHBV, PHBV/AgNPs, PHBV/AgNPs@dox, PHBV/AgNPs@flu, PHBV/AgNPs@pac, PHBV/AgNPs@gem, PHBV/AgNPs@car, PHBV/AgNPs@gin, PHBV/AgNPs@ros, PHBV/AgNPs@ore, PHBV/AgNPs@bas, and PHBV/AgNPs@thy coatings as revealed by the MTT assay. The represented data are the mean values of three independent experiments ± S.D. Statistical significance: **** *p* < 0.0001 sample vs. control.

To investigate the effect of PHBV/AgNP coatings loaded with natural compounds and anticancer drugs on the cell viability of both normal and tumor cells, the metabolic cell health was quantified by the MTT assay after 48 h and 5 days of cell-coating contact. The obtained results (Figure 11) showed that after 48 h of contact, all loaded samples impacted the cell survival of both normal and tumor cells, the cell viability being statistically significantly decreased by all coatings, independent of the drug load. Among anticancer drug-loaded coatings, PHBV/AgNPs@gem, PHBV/AgNPs@carb, and PHBV/AgNPs@dox showed the strongest potential to suppress the cell viability of the MC7 cells, while PHBV/AgNPs@pac and PHBV/AgNPs@flu exhibited a lower, but the still powerful, cytotoxic effect—reducing the cell viability by ~25% and ~33%, respectively, as compared with the untreated control. Concerning the normal cells, the anticancer-drug-loaded PHBV/AgNP coatings showed a similar cytotoxic potential, all samples triggering a statistically significant decrease of up to ~60% in the cell viability compared with the untreated control. In contrast, although there were samples loaded with natural compounds—which had effects similar to those loaded with chemotherapy compounds—PHBV/AgNPs@ros and PHBV/AgNPs@thy showed a more moderate impact on the normal cell survival, decreasing the cell viability only by ~30% compared with the untreated control. After 5 days of culture, all samples independent of the drug-loading showed a similar effect on the MCF7 cell survival rates, a dramatic decrease in the cell viability compared with the untreated control being observed in all experimental conditions. While the same pattern of cytotoxicity was maintained for most drug-loaded samples also on the normal cells, PHBV/AgNPs@ros and PHBV/AgNPs@thy did not follow this pattern. More than 70% of the cells that were in contact with the PHBV/AgNPs@ros and PHBV/AgNPs@thy coatings were viable, with lower cytotoxicity noticed in the PHBV/AgNPs@ros samples, with a significant increase in the cell viability being noticed as compared with the PHBV/AgNPs@thy coatings (**** *p* < 0.0001).

**Figure 12:**
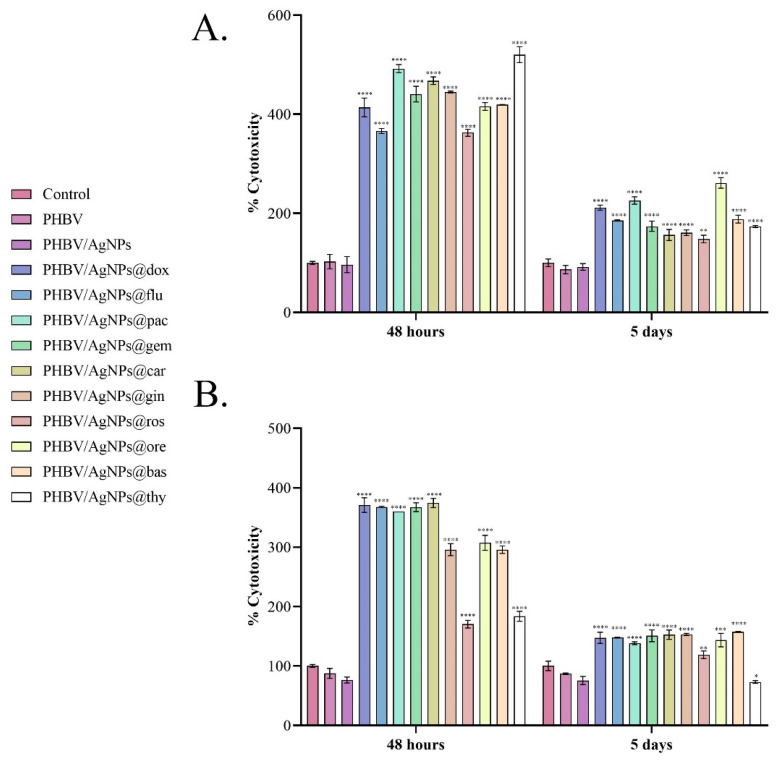
Graphical representation of uncoated samples (control) and PHBV, PHBV/AgNPs, PHBV/AgNPs@dox, PHBV/AgNPs@flu, PHBV/AgNPs@pac, AgNP PHBV/AgNPs@gem, PHBV/AgNPs@car, PHBV/AgNPs@gin, PHBV/AgNPs@ros, PHBV/AgNPs@ore, PHBV/AgNPs@bas, and PHBV/AgNPs@thy coating cytotoxicity as revealed by the LDH leakage levels quantified in culture media samples harvested from (**A**) MCF7 tumor cells and (**B**) VERO cells after 48 h and 5 days with the experimental samples. The represented data are the mean values of three independent experiments ± S.D. Statistical significance: * *p* < 0.05; ** *p* < 0.01; *** *p* < 0.001; **** *p* < 0.0001 sample vs. control.

The cytotoxic profiles revealed by the LDH assay (Figure 12) confirmed the results obtained by the MTT assay. After 48 h, in the media samples harvested from the MCF7 cell cultures in contact with the drug-loaded PHBV/AgNPs coatings, a statistically significant increase in the LDH levels compared with the experimental reference was noticed for all samples. Comparing the LDH levels in all the drug-loaded PHBV/AgNP samples, the lowest LDH levels were observed in the culture media harvested from the PHBV/AgNPs@flu samples. As fludarabine (an anticancer agent administrated to treat chronic lymphocytic leukemia) is not part of the traditional therapeutic regimen for breast cancer, the prolonged exposure of breast cancer cells to the drug could be necessary to achieve cytotoxic effects similar to those triggered by breast-cancer-specific drugs. The potential of the drug-loaded PHBV/AgNP coatings to induce MCF7 cell damage and trigger LDH release was maintained after 5 days, where all samples exhibited an increased yield in enhancing the LDH leakage. After contact with the normal cells, all drug-loaded PHBV/AgNP coatings exhibited a significant increase in the LDH levels compared with the reference, 48 h and 5 days after initiating cell–material contact. However, at both experimental times, PHBV/AgNPs@ros and PHBV/AgNPs@thy exhibited a smaller increase in the LDH levels compared with the other drug-loaded coatings, highlighting a significantly lower cytotoxic potential of these samples.

The obtained results showed that the natural-compound-loaded PHBV/AgNP coatings induced cytotoxic effects in both the tumor and the normal cells, similar to those induced by the anticancer-drug-loaded PHBV/AgNPs. Both MTT and LDH assays revealed that the cytotoxic effects were mediated by the drug payload of the PHBV/AgNP coatings, as pristine samples (PHBV and PHBV/AgNPs) did not negatively affect cell viability and cytotoxicity. Furthermore, the PHBV/AgNPs showed excellent biocompatibility with both the normal and the tumor cells, with a significant increase in cell viability being noticed when compared with the experimental reference. While the all-natural-compound-loaded PHBV/AgNP coatings significantly decreased the cell viability of the tumor cells, most of these samples showed a strong cytotoxic effect on the normal cells. However, rosemary and thyme, although they triggered a decrease in the cell viability of the normal cells, were shown to be significantly less cytotoxic compared with the other samples while still presenting the same cytotoxic effect of traditional anticancer drugs on the tumor cell viability.

#### 3.3.2. Evaluation of the Impact of Drug-Loaded PHBV/AgNPs on Cell Morphology

**Figure 13:**
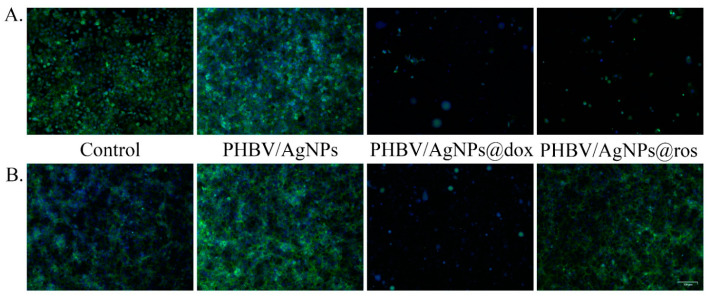
Fluorescence micrographs revealing (**A**) MCF7 cells and (**B**) VERO cells cytoskeleton after 5 days of contact with a reference sample, pristine PHBV/AgNPs, PHBV/AgNPs@dox, and PHBV/AgNPs@ros. Scale bare: 100 μm (green—actin filaments labeled with FITC-phalloidin; blue—cell nuclei stained with DAPI).

To evaluate the impact of drug-loaded PHBV/AgNPs on the normal and tumor cell morphologies, as well as the cell distribution on the material surfaces, the samples were analyzed by fluorescence microscopy after actin filaments and nuclei staining (Figure 13). The obtained results revealed that, in the absence of a drug load, all samples allowed the normal cell development of both the normal and the tumor cells, with both cell types adopting and exhibiting their typical epithelial morphologies on the surface of the coatings. Moreover, a greater proportion of cells was present on the PHBV/AgNP sample surfaces, highlighting that the coating strategy endorsed the control surface with superior biocompatibility, facilitating cell adhesion and proliferation. On this surface, both the normal and the tumor cells presented a more developed cytoskeleton as compared with the control, with a more well-defined actin expression, forming compact 3D intercellular networks, which almost covered the entire surface of the materials. The addition of either anticancer drugs or natural compounds in the PHV-AgNPs structure trigged severe alterations of the cellular architecture on both the normal and the tumor cells, except for PHBV/AgNPs@ros and PHBV/AgNPs@thy, which altered with greater affinity the tumor cells morphology. To depict these observations, an anticancer drug (doxorubicin) and a natural compound (rosemary) are selectively presented in Figure 13. The PHV-AgNPs@dox coatings induced distinct asymmetric conformational changes of actin filaments and their condensations, with both the normal and the tumor cells showing severe shrinkage after 5 days of contact with this material. Regarding the PHBV/AgNPs@ros coatings, the tumor cells exhibited a poorly developed cytoskeleton after 5 days, with a few cells scattered across the material surface. In contrast, the normal cells maintained their typical morphology in contact with the PHBV/AgNPs@ros coatings, characterized by short but well-defined actin filaments.

#### 3.3.3. Antimicrobial Activity

**Figure 14:**
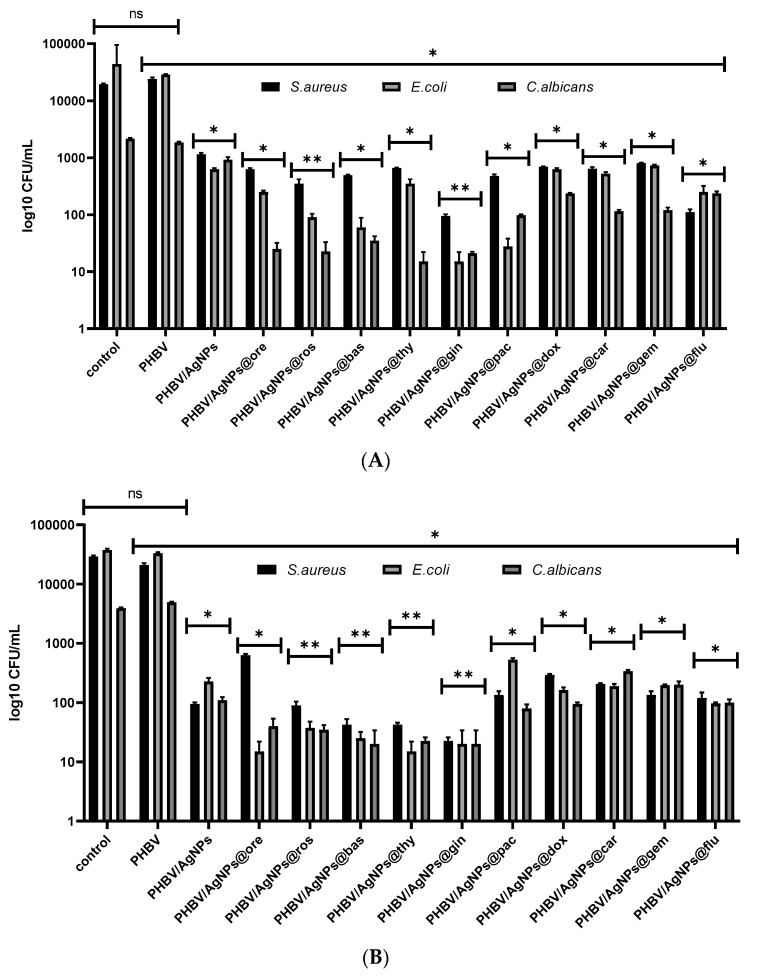
Graphical representation of log10 CFU/mL values obtained for tested microbial strains, expressing viability of bacteria incubated as 0.5 McFarland suspensions in PBS on the obtained coatings for 2 h (**A**) and 24 h (**B**). * *p* < 0.05; ** *p* < 0.001 by comparing biofilm formation on PHVB control and each AgNPs coatings, ns—not significant.

In order to analyze the antimicrobial effect of the tested coatings, several growth and viability tests in PBS were performed. Viability in PBS was assessed at two time points, i.e., 2 h and 24 h, in order to establish the bacteria-killing intrinsic potential of the nanocoated samples. It was indicated that the microbial viability was impaired in a time- and bioactive-agent-dependent manner. Significant viability loss was observed in the presence of the coatings containing the Eos, especially PHBV/AgNPs@gin, PHBV/AgNPs@ros, PHBV/AgNPs@bas, and PHBV/AgNPs@thy. The viability of these coatings was significantly impaired at both tested time points (Figure 14). Although less significant, a decreased microbial viability in the presence of the cytostatic drugs containing the coatings, especially on the PHBV/AgNPs@flu and PHBV/AgNPs@pac materials, was also observed.

**Figure 15:**
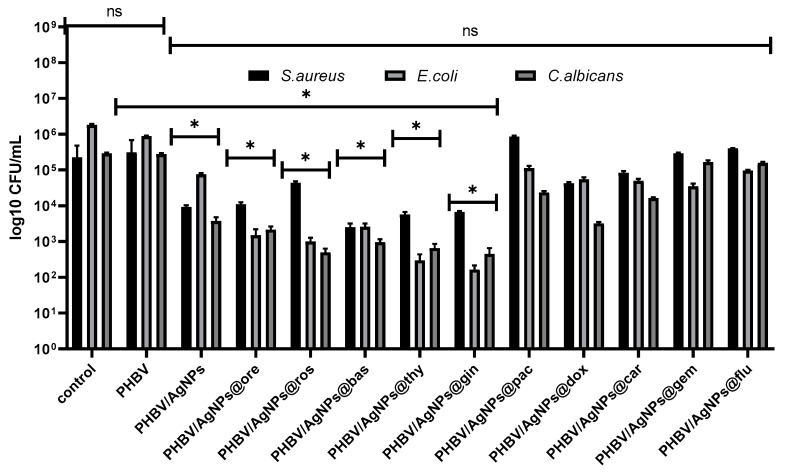
Graphic representation of log10 CFU/mL values obtained for the tested microbial strains, expressing biofilm-embedded cells developed on control and AgNPs coatings for 24 h of incubation. * *p* < 0.05; by comparing biofilm formation on PHVB control and each AgNPs coatings; ns—not significant.

Regarding the biofilm development, no significant inhibition was observed in the case of the cytostatic-containing drug coatings in the tested conditions. However, a significant biofilm inhibition was observed in all coatings containing AgNPs and EOs. Ginger and thyme EOs proved to have the highest anti-biofilm effects in all analyzed microbial strains, as revealed by Figure 15.

## 4. Discussion

The chemical composition of EOs differs significantly depending on several factors, namely the particular cultivars, the geographic area of provenience, the ecological and climate environmental conditions, the period of plant collection, and the extractive method employed.

Despite this inherent variability, the main components of the rosemary EO belong to the class of monoterpene hydrocarbons, oxygenated monoterpenes, along with sesquiterpenes and other minor compounds. According to their composition, the rosemary EOs can be divided into several chemotypes, such as α-pinene-type (α-pinene content > 20%), 1,8-cineole-type (high content of 1,8-cineole), camphor-type (camphor > 20%), and verbenone-type (verbenone > 15%) [41,42]. For instance, the GC-MS analysis of the EO extracted by hydrodistillation from a sample of the *Rosmarinus officinalis* plant material originating in the region of Shax, Tunisia, revealed 1,8-cineole as the main chemical component (23.56%) of the oil [43]. This monoterpene cyclic ether is known for its strong antimicrobial activity against several bacteria [44], which is synergistically enhanced by other minor components of the rosemary EO, such as Germacrene-D, caryophyllene, caryophyllene oxide, and terpinene-4-ol. Moreover, the main components, including 1,8-cineole as well as other components, such as camphor, α-pinene [45,46], and β-caryophyllene [47], were reported to exhibit anticancer activity. A recent extensive screening study employing rosemary EOs from six different cultivars assessed comparatively the antiproliferative effects on six cancer cell lines. All the studied rosemary EOs showed high antiproliferative ability against the human pancreatic cancer cell line SW1990 and the gastric epithelial cell line NCI-N87 [48]. The authors noticed that the same EO had different antiproliferative effects on different cancer cell lines and that different EOs with different compositions of phytochemicals had different effects on the same cancer cell line. Using a Spearman’s correlation matrix analysis, the authors of the above-cited study identified the key components responsible for the differences in antiproliferative and cytotoxic activities. It was found that camphor exhibited the highest correlation with abilities to inhibit multiple tumor cells, although it is not the most abundant component in the EOs that manifest the best antiproliferative activity. Therefore, we believe that the complex polyvalent phytochemical composition of the rosemary EO used in the present study is responsible for its observed ability to inhibit the proliferation of MCF7 breast adenocarcinoma cells. Other studies demonstrated the DNA-protective and proapoptotic effects of rosemary EO [49,50].

The antiproliferative activity of thyme EO on the MCF7 breast adenocarcinoma cell line has also been reported [51]. There are at least six chemotypes of *Thymus vulgaris* L., with different compositions of the EOs [52]. The GC-MS analysis of a sample of thyme EO isolated by hydrodistillation from a plant material native to Bosnia and Herzegovina identified the presence of 32 compounds, the most abundant among them being thymol (36.7%), *p*-cymene (30.9%), γ-terpinene (9.0%), and carvacrol (3.6%) [51]. Thymol was previously reported to exert anticarcinogenic activity on both cancer and normal cells but through different mechanisms of action. Thymol induces oxidative-stress-linked cell death, apoptotic cell death, and antiproliferative effects on cancer cells [53,54]. On the other hand, the anticarcinogenic activity of thymol in normal cells was explained by its antioxidant activity [55] and by its protective [56], antiapoptotic [55], anti-inflammatory, immunomodulatory [57], and antigenotoxic effects [58]. Thus, these literature data are in good agreement with our results regarding the selective cytotoxicity of the PHBV/AgNPs@thy nanocoatings. Regarding the other main component of the thyme EO, namely *p*-cymene, it was shown that the ruthenium (II)-*p*-cymene organometallic complex suppressed triple-negative breast cancer growth by inhibiting the tumor infiltration of regulatory T cells [59]. Furthermore, encapsulation in AgNPs increased the chemical stability and solubility, helped mitigate the intrinsic volatility, minimized the degradation of active components, enhanced the bioavailability, and supported the controlled and sustained release of the EOs. Subsequent embedment of the EO/cytostatic-drug-loaded AgNPs in the polymeric matrix of PHBV rendered our MAPLE-deposited nanocoatings highly biocompatible and biodegradable.

The antimicrobial and antibiofilm activities of the EOs have been intensively studied as an alternative to antibiotic treatment since EOs have been proven to be efficient modulators of antimicrobial resistance in various bacterial species [60,61], and their anti-adhesive effect in the early stage of the biofilm life cycle was also demonstrated [62]. In previous work, we reported on the antimicrobial and antibiofilm activities of some EOs encapsulated in microporous silica nanostructures [63]. Herein, we envisaged a synergistic effect manifested between the antimicrobial and antibiofilm properties of EO complex plant metabolite content and the intrinsic antimicrobial effect of the AgNPs, which was indeed demonstrated by the experimental data shown in Figure 14 and Figure 15.

## 5. Conclusions

We report on a comparative assessment of the cytotoxic activity against MCF7 tumor versus healthy VERO cell lines of classic cytostatic/natural essential oil (EO)-loaded silver nanoparticles (AgNPs) embedded in a biodegradable and biocompatible polymeric matrix of PHBV. AgNPs loaded with five of the most used cytostatic drugs and five EOs were first fabricated by bottom-up chemical synthesis. Subsequently, the AgNPs were embedded in the PHBV matrix, and nanocoatings of each prepared sample were deposited on Si substrates by MAPLE at a laser fluence of 400 mJ/cm^2^. The biological impact of the obtained nanocoatings on the cancer MCF7 and normal VERO cell lines was assessed by MTT and LDH assays after 2 and 5 days, respectively. The MTT assays revealed similar cytotoxicity against the MCF7 tumor cells for both PHBV/AgNPs@EOs and PHBV/AgNPs@cytostatic drugs, indicating that all EOs containing nanocoatings exhibited significant anticancer activity. One should note that PHBV/AgNPs@ros and PHBV/AgNPs@thy evidenced selective cytotoxicity on the tumor cells, while the effect on the viability of the normal cells showed a moderate impact, decreasing the cell viability by only ~30% as compared with the control, at 48 h. Moreover, after 5 days, the normal VERO cells in contact with the PHBV/Ag@ros nanocoatings showed a remarkable increase in the cell viability. Similar trends were noticed in the LDH cytotoxicity tests. Rosemary and thyme EOs containing nanocoatings showed significantly less cytotoxicity against the normal cells while maintaining a high cytotoxic effect on the cancer cells. It was observed that after 5 days of exposure to the PHBV/AgNPs@ros nanocoating, the MCF7 tumor cells exhibited a poorly developed cytoskeleton, while the healthy VERO cells preserved their normal morphology.

In addition, the antimicrobial and antibiofilm activity of PHBV/AgNPs@EOs against *S. aureus*, *E. coli*, and *C. albicans* pathogens was tested. A significant decrease in the pathogen cell viability after 2 and 24 h of exposure was observed for all tested pathogenic agents. The most efficient antimicrobial activity was demonstrated in the cases of PHBV/AgNPs@gin, PHBV/AgNPs@ros, PHBV/AgNPs@bas, and PHBV/AgNPs@thy. It is important to mention that the thyme and ginger EOs proved to have the highest anti-biofilm effects in all analyzed microbial strains.

To conclude, it was evidenced that the natural-compound-loaded PHBV/AgNP nanocoatings (i.e., PHBV/AgNPs@ros and PHBV/AgNPs@thy) preserved the same cytotoxic activity of traditional cytostatic drugs against the tumoral cells. Nevertheless, although they also triggered a decrease in the cell viability of the normal cells, this decrease was inferior to that provoked by the cytostatic drugs. The as-revealed selective cytotoxicity is a remarkable outcome of the present study and strongly recommends the newly developed nanocoatings as a valuable alternative for the treatment of breast cancer.

## Figures and Tables

**Table 1:** Sample codes of all the materials utilized and their description.

Sample Code	Description	Sample Code	Description
AgNPs@doc	silver nanoparticles functionalized with doxorubicin	AgNPs@ros	silver nanoparticles functionalized with rosemary
ANPs@car	silver nanoparticles functionalized with carboplatin	AgNPs@bas	silver nanoparticles functionalized with basil
AgNPs@flu	silver nanoparticles functionalized with fludarabine	AgNPs@thy	silver nanoparticles functionalized with thyme
AgNPs@gem	silver nanoparticles functionalized with gemcitabine	AgNPs@ore	silver nanoparticles functionalized with oregano
AgNPs@pac	silver nanoparticles functionalized with paclitaxel	AgNPs@gin	silver nanoparticles functionalized with ginger

**Table 2:** DSC-TG data related to AgNPs loaded with cytostatic drugs.

Sample	Exothermic Effects	Residual Mass (%)	Estimated Load (%)
AgNPs@gem	226/284 °C	97.97%	0.13%
ANPs@flu	229/310 °C	97.44%	0.25%
AgNPs@dox	304 °C	97.01%	0.69%
AgNPs@car	139/378 °C	97.95%	0.21%
AgNPs@pac	163/340 °C	97.86%	0.11%

**Table 3:** DSC-TG data related to AgNPs loaded with EOs.

Sample	Main Exothermic Effect (°C)	Residual Mass (%)	Estimated Load (%)
AgNPs@bas	202.2	96.97%	0.73%
ANPs@gin	201.6	97.35%	0.34%
AgNPs@ros	180.0	98.11%	0.28%
AgNPs@ore	202.6	97.62%	0.34%
AgNPs@thy	324.9	95.68%	2.05%

## Data Availability

The data can be shared up on request.
